# Late-life depression and quality of life in a geriatric evaluation and management unit: an exploratory study

**DOI:** 10.1186/1471-2318-14-77

**Published:** 2014-06-18

**Authors:** Jui-Hung Lin, Min-Wei Huang, Deng-Wu Wang, Yi-Ming Chen, Chu-Sheng Lin, Yi-Jing Tang, Shu-Hui Yang, Hsien-Yuan Lane

**Affiliations:** 1 China Medical University, Graduate Institute of Clinical Medical Science, No. 91 Hsueh-Shih Road, Taichung, Taiwan; 2 Division of Psychiatry, Chia Yi branch, Taichung Veterans General Hospital, No. 600, Sec. 2, Shixian Road, Chiayi City, West District, Taiwan; 3 Center for Geriatrics and Gerontology, Taichung Veterans General Hospital, No. 160, Section 3, Taichung-Kang Road, Taichung 40705, Taiwan; 4 Division of Allergy, Immunology and Rheumatology, Taichung Veterans General Hospital, No. 160, Section 3, Taichung-Kang Road, Taichung 40705, Taiwan; 5 Department of Family Medicine, Taichung Veterans General Hospital, No. 160, Section 3, Taichung-Kang Road, Taichung 40705, Taiwan; 6 Department of Nursing, Taichung Veterans General Hospital, No. 160, Section 3, Taichung-Kang Road, Taichung 40705, Taiwan; 7 Department of Psychiatry, China Medical University Hospital, Taichung, Taiwan

**Keywords:** Elderly, Late-life depression, Geriatric depression, Quality of life, EQ-5D, Geriatric evaluation and management unit, GEMU, ADL, GDS

## Abstract

**Background:**

Late-life depression is common among elderly patients. Ignorance of the health problem, either because of under-diagnosis or under-treatment, causes additional medical cost and comorbidity. For a better health and quality of life (QoL), evaluation, prevention and treatment of late-life depression in elderly patients is essential.

**Methods:**

This study examined (1) the differences of clinical characteristics, degree of improvement on QoL and functionality on discharge between non-depressed and depressed elderly inpatients and (2) factors associated with QoL on discharge. Four hundred and seventy-one elderly inpatients admitted to a geriatric evaluation and management unit (GEMU) from 2009 to 2010 were enrolled in this study. Comprehensive geriatric assessment including the activities of daily living (ADL), geriatric depression scale, and mini-mental state examination were conducted. QoL was assessed using the European Quality of Life-5 Dimensions and the European Quality of Life-5 Dimensions Visual Analog Scale on discharge. Information on hospital stay and Charlson comorbidity index were obtained by chart review. Chi-square tests, independent t-tests, Mann–Whitney U tests and multiple linear regressions were used in statistical analysis.

**Results:**

Worse QoL and ADL on discharge were found among the depressed. Depressive symptoms, female gender, duration of hospital stay, and rehabilitation were significant factors affecting QoL on discharge in linear regression models.

**Conclusions:**

The importance of the diagnosis and treatment of depression among elderly inpatients should not be overlooked during hospital stay and after discharge. Greater efforts should be made to improve intervention with depressed elderly inpatients.

## Background

Late-life depression (LLD) or geriatric depression is common among elderly [1,2]. However, the disorder remains under-diagnosed and under-treated [3,4]. Unlike depression in young adults, physical condition is a more heavily weighted factor in LLD [5]. In addition, comorbidity is particularly common in LLD, probably arising from biological, psychological and social mechanisms [5]. These include suicidal behavior, decreased physical, cognitive and social functioning, and greater self-neglect, all of which are associated with increased mortality [1]. The prevalence of LLD was about 10% in community, and about 40% in hospitals and long-term care facilities [2,6-9]. Functional impairment increases in patients with LLD, especially in those with multiple comorbidities [10]. Poor health status and chronic diseases are risk factors for depressive symptoms in the elderly inpatients [11]. A recent meta-analysis also showed that stroke, loss of hearing, poor eyesight, cardiac disease, and chronic lung disease were factors associated with depression in old age [12]. Despite the high prevalence of LLD, less than half of geriatric patients with the diagnosis received treatment, especially in medical settings without mental health facilities [3,4,13]. Moreover, LLD was related to a significant increase in the total cost of medical care even after adjusting for the severity of chronic medical illness [14]. Elderly inpatients who had depressive symptoms used more hospital and outpatient services than non-depressed counterparts [15]; after discharge, those who had unresolved depressive symptoms had higher rates of clinic visitation and re-hospitalization [16].

QoL is a surrogate indicator for general well-being. Wu et al. [17]. reported that QoL in elderly patients was a significant independent predictor of functional status after discharge from the hospital. Depressive symptoms are also closely related to QoL. Unsar et al. [18]. reported that depressive symptoms in elderly inpatients with chronic illness were associated with a decline in self-rated QoL. Moreover, LLD also had a significant negative impact on quality of life and was associated with increased mortality due to either suicide or chronic illness [11].

With the increasing aging population worldwide, geriatric evaluation and management units (GEMUs) have been established to meet special needs and deliver integrated care to elderly hospitalized patients [19,20]. In previous studies of GEMUs, the research has focused on functional recovery, mortality rates, readmissions, and costs of medical service among the elderly [19,21-23]. In this study, we examined (1) the differences of clinical characteristics, degree of improvement on QoL and functionality on discharge between non-depressed and depressed elderly inpatients and (2) factors associated with QoL on discharge. in a GEMU in Taiwan.

## Methods

### Study participants

**Figure 1:**
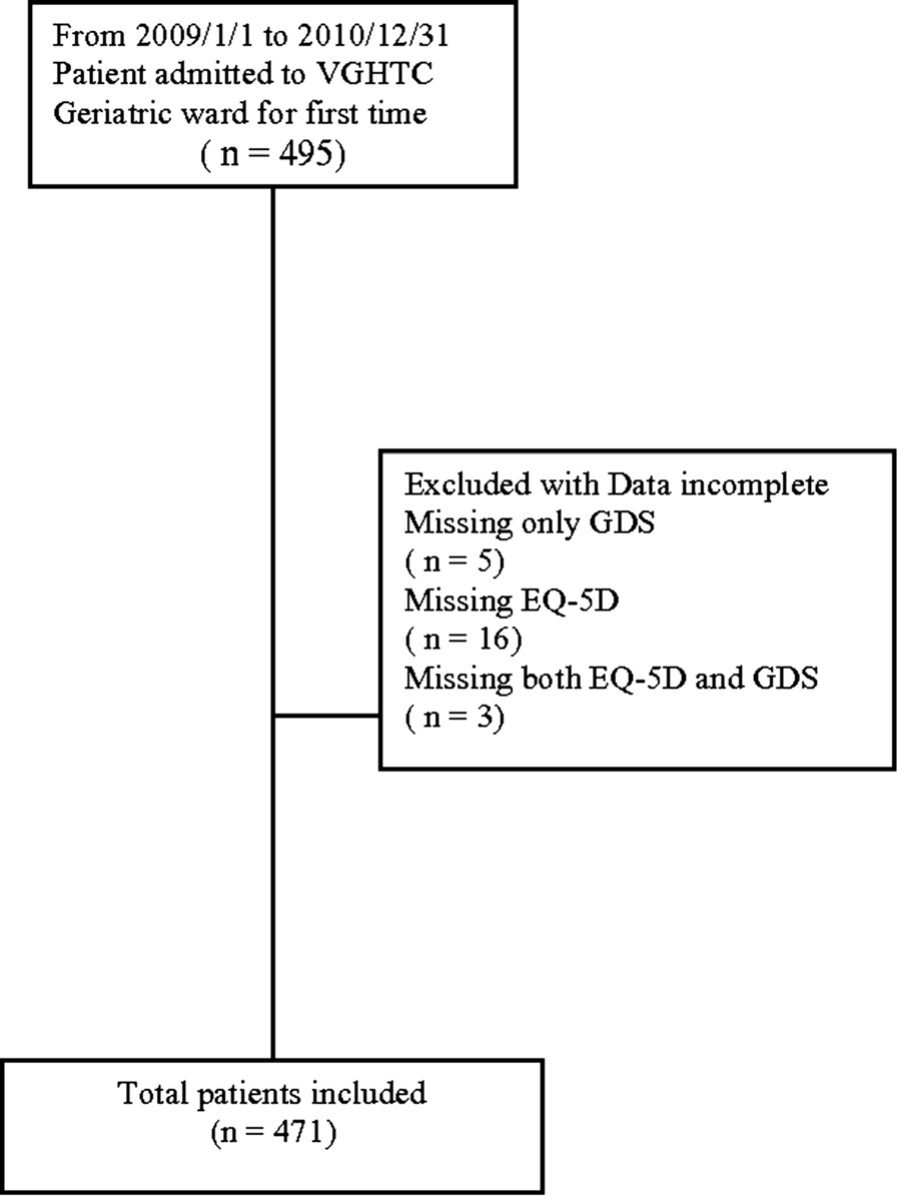
**Sampling procedures.** Flow chart of how patients included in this study.

This study was approved by The Ethics Committee of Clinical Research, Taichung Veterans General Hospital, Taiwan (file number CE11118). Four hundred and ninety-five elderly inpatients who had been hospitalized in the GEMU of Taichung Veterans General Hospital during 2009 to 2010 were included initially. After further chart review, 24 patients were excluded because their data were incomplete. A total of 471 patients were finally used in the statistical analysis. The sampling procedures are shown in Figure 1.

### Study setting

The GEMU in Taichung Veterans General Hospital is an 18-bed hospital facility devoted to the care of frail elderly patients with acute functional decline in a tertiary teaching hospital in central Taiwan. A nearby rehabilitation room for onsite physiotherapy and occupational therapy, along with visual calendars and clocks, provides accessibility for activities related to physical enablement and cognitive integrity. The staff of the GEMU consisted of two geriatricians, one case manager, one rehabilitation specialist, one psychiatrist, and two residents from the department of internal medicine and family medicine. The nurse members of the GEMU received formal training in geriatric nursing and skills for elderly care. The core members of the multi-disciplinary team in the GEMU included a physiotherapist, occupational therapist, dietitian, social worker, pharmacist, and psychologist. Meetings were arranged once a week to report the assessments of different specialists, set goals, discuss various problems, and plan discharge. In general, rehabilitation in the GEMU aimed at improving ADL, walking independence, muscle strengthening, and balance training. Tailor-made rehabilitation plans were implemented by physiotherapists, occupational therapists, and nurses to ensure mobilization throughout the day and during holidays.

### Comprehensive geriatric assessment

All patients in this study completed the comprehensive geriatric assessment (CGA). The CGA includes basic personal information (age, gender, history of chronic illness, education, source of referral) and various assessment tools, comprising the activities of daily living (ADL), geriatric depression scale (GDS), mini-mental state examination (MMSE), and European Quality of Life instrument-5 Dimensions (EQ-5D)/European Quality of Life instrument-5 Dimensions Visual Analog Scale (EQ-5D VAS). All assessments were conducted on admission and discharge.

ADL were evaluated using the Barthel Index (BI), which consists of 10 categories including eating, toileting, personal hygiene, dressing, walking, and climbing stairs [24]. The MMSE was used as a screening tool for cognitive integrity [25]. A 15-item geriatric depression scale (GDS, range 0–15) was used as a screening tool for depressive symptoms [26,27], and higher scores indicated more perceived symptoms. The EQ-5D is a questionnaire composed of five dimensions (mobility, self-care, usual activities, pain/discomfort, and anxiety/depression) for self-rated quality of life [28]. Levels of severity in each dimension are defined as no problems (one point), some problems (two points), and major problems (three points). The total summed score of EQ-5D ranges from five to 15. Higher scores in the EQ-5D mean poorer self-rated quality of life. The EQ-5D VAS is a 20-cm visual analog scale for patients to mark their current health status [29]. Higher scores in the EQ-5D VAS represent better self-rated health status/quality of life (scores from 0 to 100).

Chart review was also performed for information about the hospital stay. A list of chronic illnesses was recorded, including hypertension, diabetes mellitus, cerebral vascular disease, chronic renal disease, chronic obstructive pulmonary disease, atrial fibrillation, arthritis and gout. Comorbid conditions were measured using the Charlson comorbidity index (CCI) [30].

### Statistical analysis

Data in the text and tables are expressed as means ± S.D. The Statistical Package for the Social Sciences (SPSS) version 10.0 software (SPSS Ltd., Chicago, IL, USA) was used to perform all statistical analyses. Categorical variables were compared using the Chi-square tests. Independent sample t-tests and Mann–Whitney U tests were used for comparisons between continuous variables as appropriate. Multiple linear regression analysis was used to identify independent factors for QoL. All tests with p < 0.05 were considered statistically significant.

## Results

**Figure 2:**
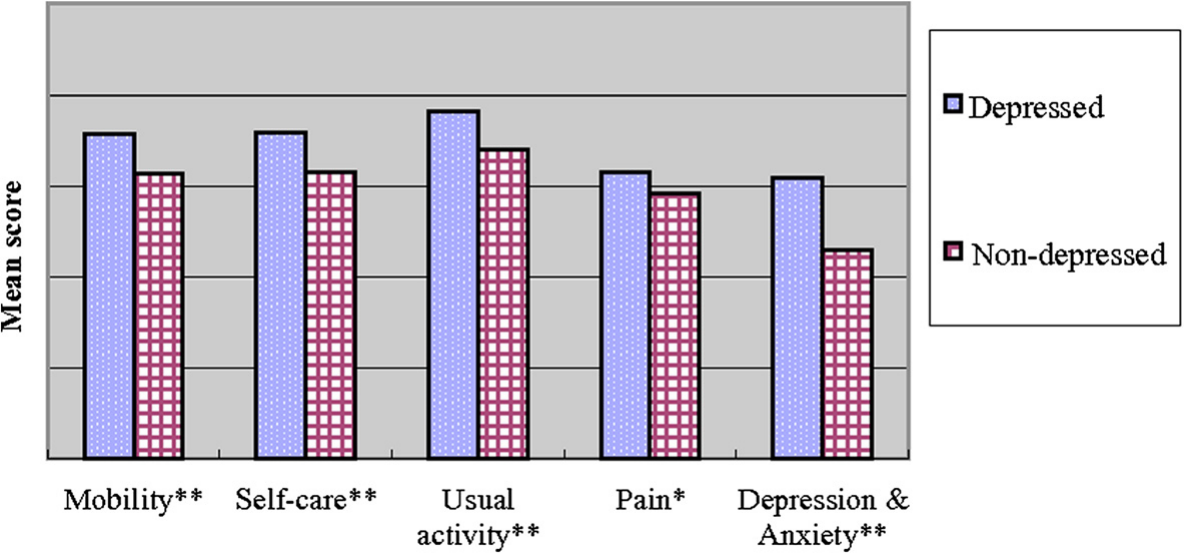
**Comparison of EQ-5D subscales on discharge.** The subscales of EQ-5D are shown as Mobility (p < 0.001); Self-care (p = 0.005); Usual activity (p = 0.003); Pain (p = 0.023); Depression and Anxiety (p < 0.001). Mann–Whitney *U* test. (**) p < 0.01. (*) p < 0.05.

**Figure 3:**
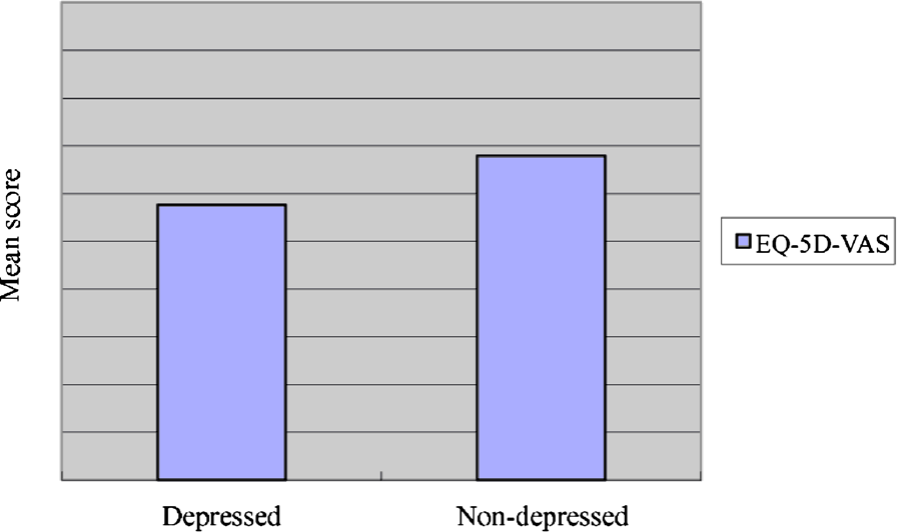
**Comparison of EQ-5D-VAS on discharge.** Significant difference was identified when comparing EQ-5D-VAS between depressed and non-depressed elderly inpatients applying Mann–Whitney *U* test.

Comparisons between the non-depressed and depressed group are summarized in Table 1. The prevalence of depressive symptoms was 54.4% in the elderly patients. There were no differences in age (80.79 ± 5.98 vs 79.82 ± 6.30), gender (71.5% male vs 74.9% male), education (omitted) and length of hospital stay (13.61 ± 9.18 vs 12.75 ± 9.62) between the non-depressed and depressed group. Minimal differences were seen in the sources of referral to the GEMU in the two groups (p = 0.042). There was no difference in the severity of chronic illness as represented by similar Charlson comorbidity indices (p = 0.986). MMSE and ADL showed significant differences between the two groups on admission (p values <0.001). Depressed elderly inpatients showed more cognitive impairment and worse ADL scores on admission; worse ADL scores and less satisfaction in QoL were shown on discharge (EQ-5D, EQ-5D-VAS).Analysis of the subscales of EQ-5D showed significant differences in each subscale between the two groups (Mobility: p < 0.001; Self-care: p = 0.005; Usual activity: p = 0.003; Pain: p = 0.023; Depression and Anxiety: p < 0.001) (Figure 2). A significant difference was also noted in EQ-5D-VAS on discharge (p < 0.001) (Figure 3). The elderly inpatients with depressive symptoms on admission showed worse QoL on discharge.

**Table 1:** Total demography data and sub-groups comparison (with/without depressive symptoms)

**Paraemeters**	**Total**	**Depressed (GDS ≧ 5)**	**Non-depressed (GDS < 5)**	**Compare depressed and non-depressed**
	**n = 471**	**n = 256**	**n = 215**	
**Basic information**	**Mean ± S.D. or n (%)**	**Mean ± S.D. or n (%)**	**Mean ± S.D. or n (%)**	**p value**
Age	80.35 ± 6.14	80.79 ± 5.98	79.82 ± 6.30	0.089^(a)^
Gender (Male)	344 (73.0)	183 (71.5)	161 (74.9)	0.408^(c)^
Education				0.438^(b)^
Below elementary school	142 (30.1)	77 (30.1)	65 (30.2)	
Elementary school	144 (30.6)	82 (32.0)	62 (28.8)	
Junior high school	57 (12.1)	25 (9.8)	32 (14.9)	
Senior high school	76 (16.1)	45 (17.6)	31 (14.4)	
Above college	52 (11.0)	27 (10.5)	25 (11.6)	
Refer source				0.042^(b)^
General ward	127 (27.0)	58 (22.7)	69 (32.1)	
Out patient clinic	199 (42.3)	122 (47.7)	77 (35.8)	
Emergency department	120 (25.5)	64 (25.0)	56 (26.0)	
Psychiatric department	25 (5.3)	12 (4.7)	13 (6)	
Hospital stay (Days)	13.22 ± 9.38	13.61 ± 9.18	12.75 ± 9.62	0.325^(a)^
Charlson Comorbidity Index	3.65 ± 2.97	3.66 ± 3.00	3.62 ± 2.96	0.986^(c)^
On admission				
GDS on admission	5.65 ± 3.76	8.36 ± 2.73	2.35 ± 1.29	<0.001^(c)^
MMSE on admission	21.99 ± 5.77	21.03 ± 5.53	23.13 ± 5.86	<0.001^(c)^
ADL on admission	61.94 ± 31.57	57.68 ± 31.03	67.01 ± 31.53	<0.001^(c)^
On discharge				
ADL on discharge	69 ± 30.13	65.21 ± 30.15	73.51 ± 29.55	<0.001^(c)^
EQ-5D on discharge	8.09 ± 2.59	8.61 ± 2.58	7.46 ± 2.46	<0.001^(c)^
EQ-5D-VAS on discharge	62.32 ± 21.89	57.58 ± 21.25	67.96 ± 21.33	<0.001^(c)^

^(a)^Independent-Sample *T* Test a = 0.05.

^(b)^Chi-Square Test.

^(c)^Mann–Whitney *U* Test.

Multiple linear regressions were performed for factors that were significantly associated with QoL on discharge (from Table 1). The results are summarized in Table 2. Depressive symptoms, age, female gender, duration of hospital stay, and in-hospital rehabilitation were significant factors associated with EQ-5D score on discharge (adjusted R^2^ = 0.24 in enter mode and 0.25 in stepwise mode).

**Table 2:** Multiple linear regression: Factors that significant affect quality of life on discharge

	**EQ-5D on discharge**							
	**Multiple linear regression (enter)**				**Multiple linear regression (stepwise)**			
	**S.E.**	**t**	**Regression coefficient**	**p value**	**S.E.**	**t**	**Regression coefficient**	**p value**
(Constant)	1.46	0.17		0.866	1.46	0.23		0.82
Age	0.02	3.44	0.15	0	0.02	3.59	0.15	<0.001
Female	0.25	3.24	0.14	0.001	0.25	3.17	0.13	0
CCI	0.04	0.92	0.04	0.356				
Hospital stay	0.01	6.53	0.27	<0.001	0.01	6.52	0.27	<0.001
Polypharmacy	0.24	1.34	0.06	0.179				
Depressed (GDS ≥ 5)	0.21	3.67	0.15	<0.001	0.21	3.83	0.16	<0.001
Rehabilitation	0.23	5.91	0.25	<0.001	0.23	6.32	0.26	<0.001
Adj. R^2^		0.24				0.25		

## Discussion

### Late-life depression in GEMUs

A study in the United Kingdom demonstrated depression symptoms among 44% of elderly inpatients in district general hospitals (GDS ≥ 5) [31]. We found a higher prevalence of depressive symptoms among Chinese elderly inpatients in our GEMU using the same research criteria. There has been no published literature explaining why there should be a higher prevalence of depression symptoms in GEMUs. The difference might lie in the components of a GEMU setting. Our GEMU provides services in a tertiary medical center and receives referrals from primary care and district general hospitals. A previous study has shown that elderly patients with physical illnesses were more likely to suffer from depression than those without [12]. In addition, elderly inpatients who are depressed appear to have a very poor prognosis: the recovery rate among these patients is low and the mortality rate high [32]. Although it is inclusive if depression predicts mortality in elderly inpatients, the disorder does increase the chance of disability and is associated with worsened outcomes of comorbid chronic medical diseases [33,34]. One possible reason that the primary care units and district general hospitals referred their patients to our GEMU was that their patients had more complicated health problems. However, our further statistical analysis showed no significant difference in Charlson comorbidity indices between depressed and non-depressed elderly patients, suggesting that chronic physical illness was not a determinant in these patients. Some non-physical health-related factors may also contribute to depression in the elderly [5]. Our data were unable to provide answers about these possibilities.

Late-life depression has been shown to lead to further functional and cognitive decline [4]. In our study, depressed elderly inpatients showed more cognitive impairment and worse ADL on admission compared with non-depressed patients. Furthermore, depressed elderly patients had little improvement in ADL on discharge compared with non-depressed patients.

### Quality of life

Depressed elderly inpatients in our GEMU had less satisfaction and QoL in our study. Depressive symptoms, age, female gender, duration of hospital stay, and in-hospital rehabilitation were significant factors that affected QoL on discharge in a multiple linear regression model. The results were compatible with previous non-GEMU studies. For example, McCall et al. reported that age and severity of depression affect the quality of life in depressed inpatients [35]. Doraiswamy et al. reported late-life depression, female gender, and advanced age were associated with poor quality of life [36].

Saltvedt et al. indicated that to improve QoL, a GEMU should emphasize an integration of interdisciplinary assessments of all relevant disorders, prevention of complications and iatrogenic conditions, early mobilization/rehabilitation, and comprehensive discharge planning [37]. Phibbs et al. showed that specialized and individualized inpatient rehabilitation performed in the GEMU improved treatment outcomes and decreased the risk of living in a nursing home [38-41].

In addition, compared with previous rehabilitation studies in settings other than a GEMU, our GEMU had much shorter durations of hospital stay. More time for rehabilitation to take effect may be needed for better outcomes and QoL. Also, exercise in depressed elderly patients has shown effects on improvement of depression and quality of life [42-44].

### Advantage of this study and limitations

The major advantage of this study was the use a comprehensive set of assessment tools for answering the questions of QoL and functionality in elderly patients. The implications of this study are also limited. The main concern was the short duration of hospital stay which restricts further understanding of the role of the factor. Second, how Taiwan people interpret illness or impairment occurred on elderly is unclear. The cultural factor may somewhat impacts levels of self-rated health in QoL or depression.

## Conclusion

A high prevalence of depressive symptoms in our GEMU was found. The diagnosis and treatment of late-life depression should be emphasized in elderly inpatients since quality of life and functional recovery on discharge would be impacted negatively by the failure to detect and remediate this condition. Because of the tertiary medical center policy of limited duration of hospital stays, more efforts should be made to develop better brief interventions with depressed elderly inpatients. We suggest that management of depressive symptoms must be continued from the hospital stay to the community to reduce medical costs and improve quality of life.

## Abbreviations

ADL: Activity of daily living; BI: Barthel index; CCI: Charlson comorbidity index; CGA: Comprehensive geriatric assessment; EQ-5D: European quality of life instrument-5 dimensions; EQ-5D VAS: European Quality of life instrument-5 dimensions visual analog scale; QoL: Quality of life; GDS: Geriatric depression scale; GEMU: Geriatric evaluation and management unit; LLD: Late-life depression; MMSE: Mini-mental state examination.

## Competing interests

This was not an industry supported study. The authors have indicated no competing interest.

## Authors’ contributions

JHL conceived of the study, and participated in its design and coordination and helped to draft the manuscript. MWH, HYL and DWW contributed to statistical analyses and interpretation of the results. YMC, CSL, YJT and SHY participated in clinical assessments and chat review of the patients. All authors revised the manuscript and approved the final manuscript.

## Pre-publication history

The pre-publication history for this paper can be accessed here:

http://www.biomedcentral.com/1471-2318/14/77/prepub
